# Sulfonated graphene oxide impregnated cellulose acetate floated beads for adsorption of methylene blue dye: optimization using response surface methodology

**DOI:** 10.1038/s41598-022-13105-4

**Published:** 2022-06-04

**Authors:** Islam K. Basha, Eman M. Abd El-Monaem, Randa E. Khalifa, Ahmed M. Omer, Abdelazeem S. Eltaweil

**Affiliations:** 1 Chemistry Department, Faculty of Science, Alexandria University, Alexandria, 11432 Egypt; 2 Polymer Materials Research Department, Advanced Technology and New Materials Research Institute (ATNMRI), City of Scientific Research and Technological Applications (SRTA-City), P. O. Box: 21934, New Borg El-Arab City, Alexandria Egypt

**Keywords:** Composites, Pollution remediation

## Abstract

New multi-featured adsorbent beads were fabricated through impregnation of sulfonated graphene (SGO) oxide into cellulose acetate (CA) beads for fast adsorption of cationic methylene blue (MB) dye. The formulated SGO@CA composite beads were thoroughly characterized by several tools including FTIR, TGA, SEM, XRD, XPS and zeta potential. The optimal levels of the most significant identified variables affecting the adsorption process were sequential determined by the response surface methodology (RSM) using Plackett–Burman and Box–Behnken designs. The gained results denoted that the surface of SGO@CA beads displayed the higher negative charges (− 42.2 mV) compared to − 35.7 and − 38.7 mV for pristine CA and SGO, respectively. In addition, the floated SGO@CA beads demonstrated excellent floating property, fast adsorption and easy separation. The adsorption performance was accomplished rapidly, since the adsorption equilibrium was closely gotten within 30 min. Furthermore, the adsorption capacity was greatly improved with increasing SGO content from 10 to 30%. The obtained data were followed the pseudo-second order kinetic model and agreed with Langmuir adsorption isotherm model with a maximum adsorption capacity reached 234.74 mg g^−1^. The thermodynamic studies designated the spontaneity and endothermic nature of MB dye adsorption. Besides, the floated beads exposed acceptable adsorption characteristics for six successive reuse cycles, in addition to their better adsorption selectivity towards MB dye compared to cationic crystal violet and anionic Congo red dyes. These findings assume that the formulated SGO@CA floated beads could be used effectively as highly efficient, easy separable and reusable adsorbents for the fast removal of toxic cationic dyes.

## Introduction

Water pollution is considered one of the most important critical problems facing countries, since wastewater poses a significant threat to the ecosystem as well as human health^1^. One source of contamination is dye discharge from industries such as textile, paper, leather tanning, food processing, plastics, cosmetics, rubber, printing and dye manufacturing^2–4^. Methylene blue (MB) is a highly stable cationic dye which readily aggregates and highly soluble even at minimal concentrations causing harmful impacts^5–8^. Therefore, revelation to MB can lead to breathing difficulties, eyes burn, vomiting, nausea, and mental bewilderment^9^. Various techniques have been employed for dyes remediation from contaminated water such as advanced oxidation, membrane filtration, photocatalytic degradation, and adsorption^10–14^. Adsorption is recognized one of the most promising treatment technologies for the removal of various harmful contaminants from wastewater owing to its excellent features comprising higher efficacy, low-cost production and simple processing^4,15–17^. Cellulose is the most abundant natural a polysaccharide polymer. Cellulose has a number of advantages including low cost, biodegradability and renewability^18^. More importantly, cellulose is environmentally friendly, as it can easily be decomposed by certain decomposers and returned to the natural carbon cycle^19^. However, cellulose is insoluble in water and most organic solvents, and it has low reactivity, making it difficult to modify directly to create other usable materials^20^. To overcome this obstacle, commercially available cellulose derivatives, such as cellulose acetate (CA) and carboxymethyl cellulose (CMC) have been utilized^21,22^. Cellulose acetate (CA) is a remarkable cellulose ester in industry due to its favorable physical properties which is abundant natural sources, good biodegradability, low cost and easy processing^23^. The active functional groups of CA facilitate its modifications with other functional groups for improving its adsorption characteristics^24^. Therefore, numerous studies focused on modification techniques of CA such as grafting, composite formation through incorporation of MOFs, carbon-based materials and active metal oxides^25–27^.

Graphene oxide (GO) is produced by the oxidation of graphene, containing abundant oxygen-functional carbonyl, epoxide, hydroxyl and carboxyl groups^28–30^. These active groups are responsible for improvement its chemical reactivity compared with original graphene as well as simplify its physical and chemical modifications^31^. Moreover, the large specific surface area, hydrophilicity and high negative charge density of graphene supported its potential application in the field of water pollution control. This is due to its coordinate aptitude and electrostatic interactions with various pollutants such as heavy metals, herbicides, inorganic anions and organic dyes from aquatic environment^32^. Nevertheless, GO tends to aggregate in aquatic environments via the hydrophobic effect because of strong inter planar interactions of π–π key^33^. Correspondingly, the surface area of GO will be reduced, and the adsorption efficiency will be conversely affected. Combination of GO or its functionalized derivatives with polymeric carriers such as chitosan, cellulose and alginate can efficiently overcome the obstacles of GO as well as facilitate its separation process from the adsorption media^34,35^.

Besides, the experimental design is a powerful tool for identifying the interactions and relativistic importance of several factors^36^. For optimizing complex processes, the employment of statistical designs such as Plackett–Burman (PB) and Box–Behnken (BB) can produce remarkable results. Response surface methodology (RSM) is a statistical technique that entails factorial design and regression analysis. It is used to determine the relative relevance of individual elements and their interacting controls^37^. The most often used response surface approaches are central composite and Box-Behnken^38^. Additionally, RSM can be used to reduce the number of laboratories experiments necessary to estimate various variables and their interactions. RSM is a particularly effective technique for this research since it generates statistical patterns that may be used to deduce the association between the optimized variables^39^.

Till now, there are no studies involving the fabrication of CA floated beads and their composites for adsorption of toxic dyes, whereas adsorbents-based cellulose acetate have developed mainly in forms of films and nanofibers. Accordingly, an attempt in this study was made to construct efficient SGO@CA floated beads for adsorptive removal of cationic MB dye. The negativity charged functional groups of SGO are expected to boost the adsorption capacity of the developed beads via the strong electrostatic interaction with the positively charged methylene blue dye. Moreover, the floating ability of SGO@CA beads assist to easy separation of adsorbent from pollutant media without centrifugation or any filtration technique. The developed floated beads were characterized using several characterization tools. The optimum levels of the most noteworthy identified variables were determined using a response surface methodology. A complete batch adsorption studies were conducted under different adsorption conditions. In addition, reusability and adsorption selectivity were evaluated. Besides, the possible adsorption mechanism of MB dye onto SGO@CA floated composite beads was also suggested.

## Experimental

### Materials

Cellulose acetate (M.wt. 30,000), graphite powder, dimethyl sulfoxide (DMSO), Methylene blue tri-hydrate (MB), Crystal violet (CV) dye, Congo red (CR) dye and Hydrogen peroxide (H_2_O_2_) were purchased from Sigma-Aldrich Co. (Germany). Potassium permanganate (KMnO4), methylene chloride (CH_2_Cl_2_), chlorosulfonic acid (ClSO_3_H), sulfuric acid (H_2_SO_4_), Nitric acid (HNO_3_), hydrochloric acid (HCl), acetone and Ethanol were provided from Aladdin Reagent Co., Ltd (China).

### Synthesis of graphene oxide

Graphene oxide (GO) was synthesized by the modified Hummers method^40^. Primarily, fuming nitric acid, concentrated sulfuric acid and potassium permanganate added slowly to graphite powder with ratio 50:1, 15:1 and 6:1, respectively. The mixture was ultra-sonicated and stirrers at below 40 °C for complete oxidation stage. Finally, the mixture poured in distillated water after that hydrogen peroxide and hydrochloric acid added to remove any impurities of unreacted manganese from GO solution. The obtained GO was washed with distillated water to reach pH almost 6.

### Preparation of sulfonated graphene oxide (SGO)

Accurate 2 g of GO powder was dispersed in sonicated and stirrer in 50 mL of methylene chloride (CH_2_Cl_2_) for dispersion the powder in solution. Thereafter, 10 mL chlorosulfonic acid (ClSO_3_H) added to slurry solution under reflux conditions overnight to complete sulfonating process of graphene oxide. Finally, water added slowly to slurry solution to minimize evaporation intensive vapor of hydrochloric gas^41^. After stopping the reaction by water, the slurry solution was washed by acetone and water in centrifuge system.

### Formulation of SGO@CA floated beads

**Figure 1:**
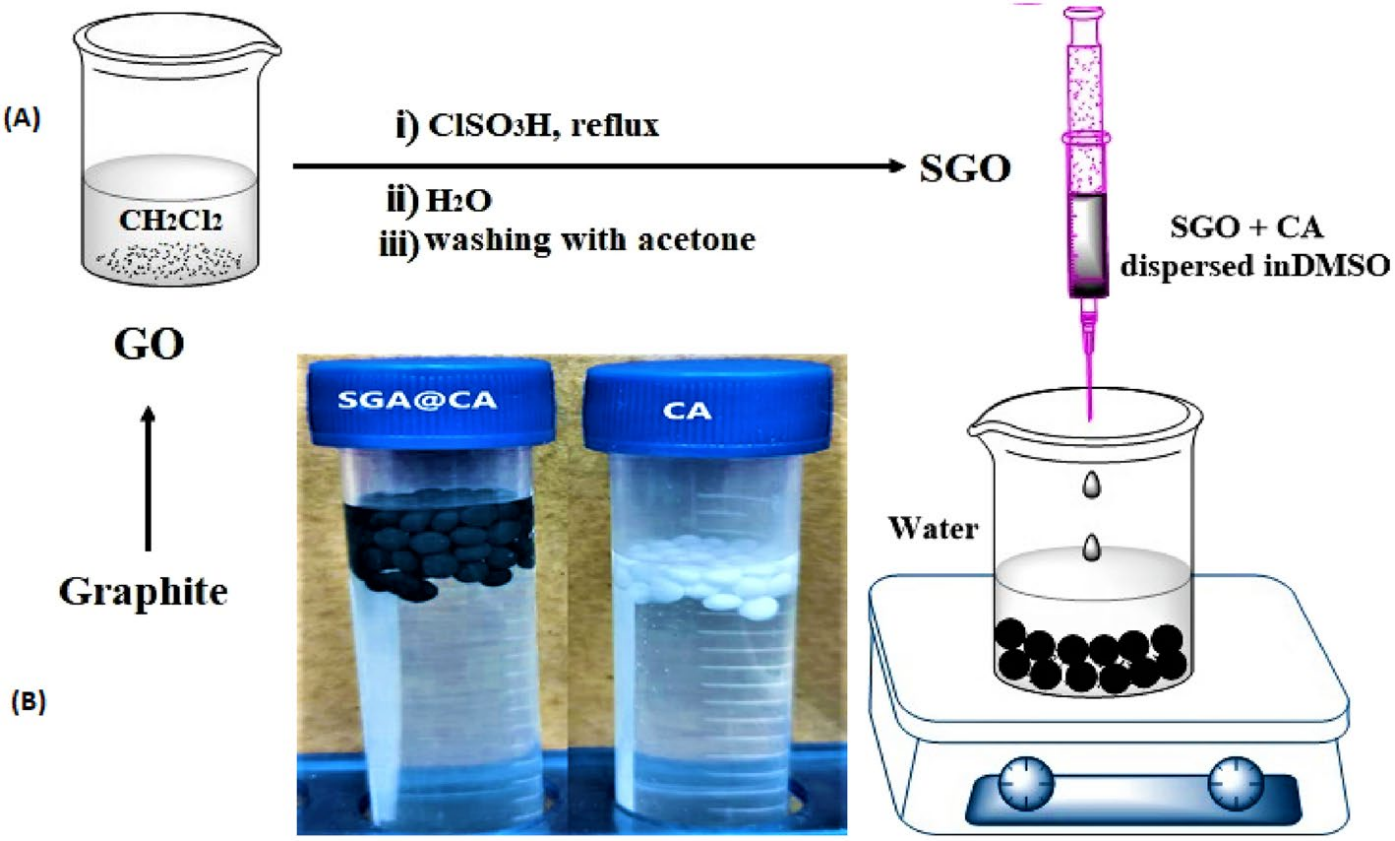
(**A**) A schematic representation for the formulation of SGO@CA floated beads, (**B**) laboratory images for freshly prepared SGO@CA floated beads.

Cellulose acetate (10%) was dissolved in DMSO followed by addition of 5–30% (wt) of SGO to solution. The solution mixture was left for 1 h under continuous stirring at room temperature to gain a homogenous mixture. Floated beads were formulated by the solvent exchange technique via dropping the composite solution in water media using a plastic syringe (3 cm^3^). Finally, the obtained SGO@CA floated beads were collected and dried at room temperature. A schematic diagram describes the formulation of SGO@CA floated beads and laboratory images for CA and its composite floated beads were displayed in Fig. 1.

### Characterization

The chemical structure of the developed composite beads was investigated by Fourier Transform Infrared Spectroscopy (FTIR, Nicolet 6700 spectrometer, Japan). The thermal characteristics were analyzed by Thermal Gravimetric Analyzer (TGA, Model 50/50H, Shimadzu, Japan). Moreover, the surface morphologies were investigated by a Scanning Electron Microscope (SEM, Hitachi Limited, Japan,), while the crystal phases were inspected by X-ray Diffractometer (XRD; MAC Science M03XHF). Zeta Potential (ZP; Malvern) and X-ray photoelectron spectroscopy (XPS, Thermo Scientific ESCALAB 250Xi VG, USA) were employed to examine the surface charges and the elemental compositions of the developed adsorbent, respectively.

### Plackett–Burman design

Variables affecting the MB adsorption capacity of SGO@CA composite beads were tested for screening purposes. Low and high values at two levels (− 1 and + 1) for each component were evaluated using the Plackett–Burman design^39^. The studied factors and degrees of each component employed in experimentation were depicted in Table S1. The first-order model is the foundation for Plackett–Burman experimental design.

1 $$Y={\beta }_{0}+\sum {\beta }_{i }{X}_{i}$$

where *Y*,* β*_0_,* β*_*i*_, and *x*_*i*_ is the MB adsorption capacity (response), the model intercepts, the linear coefficient, and the level of the independent variable value, respectively.

A total of twelve experiments were conducted to examine the effects of five different variables. The mean MB adsorption capacity was used as a response in all three experiments. Table S2 displayed the project's design matrix, while multiple regression analysis in Microsoft Excel was used to examine the Plackett–Burman trial's outcomes.

### Box–Behnken design

Box-Behnken design was used to clarify the nature of the RSM in the experimental zone (Table S3). Variables with a positive influence and the greatest confidence levels were classified into three categories, denoted by the codes − 1, 0 and + 1 for low, middle, and high values, respectively. The design matrix for a 27 trials experiment is shown in Table S4, while values of estimated regression coefficient and corresponding t and P value were stated in Table S5. A second-order polynomial function was fitted to the connection between independent factors and response to predict the best response. The equation that employed for the four variables can be expressed as follows:

2 $$Y={\beta }_{o}+{\sum }_{i=1}^{k}{\beta }_{i}{X}_{i}+{\sum }_{i=1}^{k}{\beta }_{ii} {X}_{i }{X}_{i}+\sum {\sum }_{i<j}{\beta }_{ij}{X}_{i}{X}_{j}$$

where *Y* is the response, *β*_*0*_*, β*_*i*_*, β*_*ii*_*, and β*_*ij*_ are the constants coefficient of the intercept term, linear term, quadratic term and interaction term, respectively. *x*_*i*_ and *x*_*j*_ are coded independent variable.

The mean MB adsorption capacity was used as a response in the experiments, which were carried out in triplicate. The coefficient R^2^ demonstrated the polynomial model equation's fit quality. The JMP algorithm was used to determine the best value for dye adsorption capacity using SGO@CA composite beads. The concurrent effects of the four most significant independent variables on each response were depicted using a three-dimensional graph created using Statistica7.0.

### Batch adsorption studies

The adsorption of MB dye experiments was achieved by batch experiments. In brief, known amounts of dried SGO@CA composite beads (0.005–0.025 g) were thoroughly added to 50 mL of initial concentration of MB dye solutions (50–300 mg L^−1^). The adsorption medium pH was adjusted in the range of pH 3–11, while adsorption temperature was varied from 25 to 45 °C. Next, samples were collected after time intervals and filtered regularly, while the residual MB concentration was assayed at wavelength 664 nm using UV-spectrophotometer. The removal (%) and adsorption capacity (q) were calculated according to the following equations^42,43^:

3 $$\mathrm{R \%}=\frac{{\mathrm{C}}_{0 }-{\mathrm{C}}_{\mathrm{t}}}{{\mathrm{C}}_{0}} \times 100$$

4 $${\mathrm{q}}_{\mathrm{e}}=\frac{{(\mathrm{C}}_{0}-{\mathrm{C}}_{\mathrm{t}})\times \mathrm{V}}{\mathrm{w}}$$

where *C*_*0*_ and *C*_*t*_ signify the initial concentration of MB dye and at a definite time *t*, respectively. *W* and *V* are the weight of composite beads and the volume of MB dye solution, respectively.

### Regeneration and selectivity studies

To examine the aptitude of the fabricated SGO@CA composite beads to reuse, a regeneration test was performed for six consecutive adsorption–desorption cycles. After the completion of the adsorption process, the dye-adsorbed composite beads were gently separated from the batch adsorption medium and subsequently immersed in a solution of the desorption medium (1M NaCl/methanol), while the process was conducted under gentle stirring for 1 h. Later, the dye-adsorbed beads were separated from the desorption medium and reused for several repeated adsorption cycles. Besides, adsorption selectivity was examined by employing anionic Congo red dye in presence of cationic MB dye.

## Results and discussion

### Characterization of SGO@CA beads

#### FTIR

**Figure 2:**
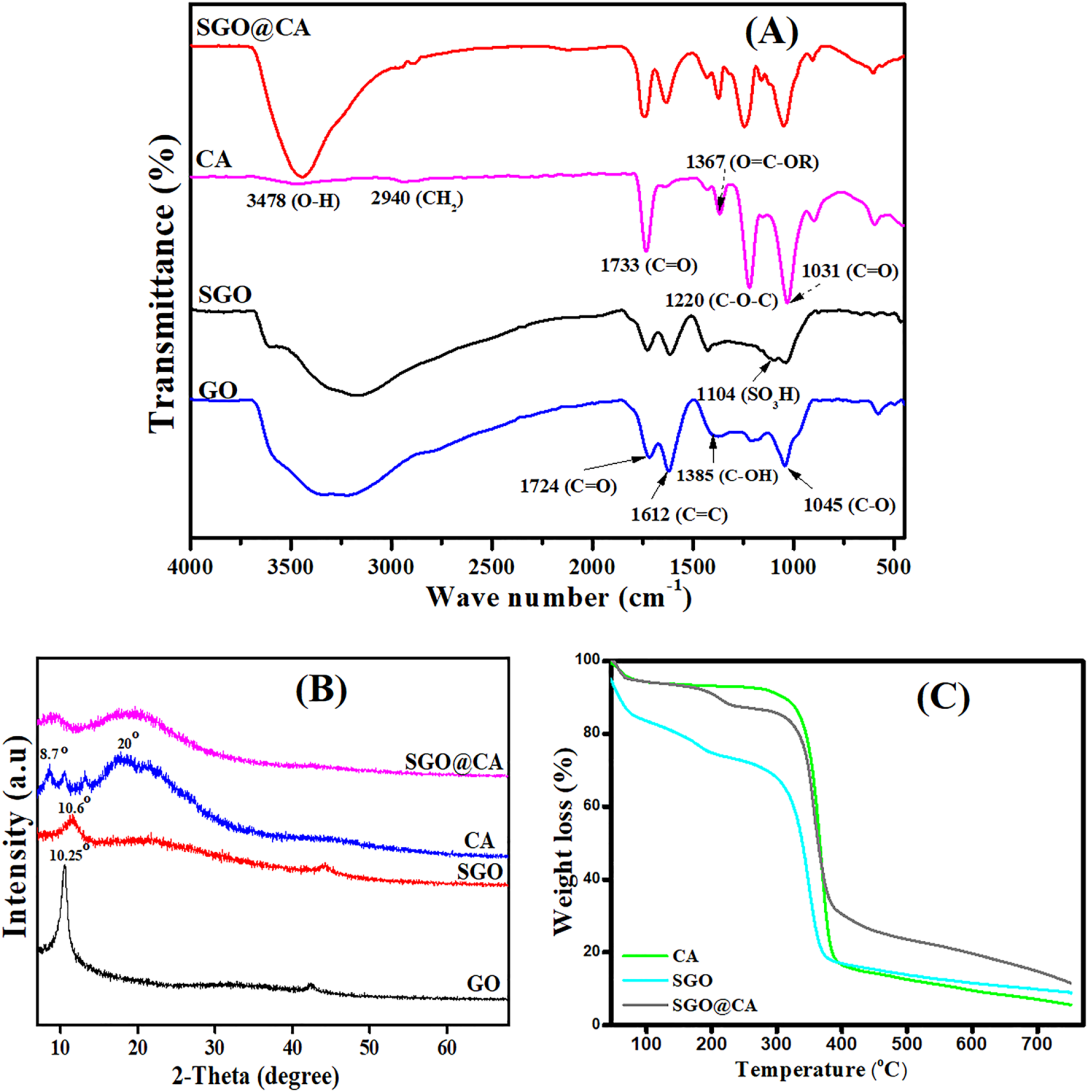
(**A**) FTIR, (**B**) XRD and (**C**) TGA of SGO@CA composite beads and their pristine components.

Figure 2A represents FTIR spectra of GO, SGO, CA and SGO@CA beads. FTIR spectrum of GO reveals its characteristic absorption bands at 1045, 1724, 1612 and 1385 cm^−1^ that are attributed to epoxy C–O, C=O, C=C and C–OH, respectively^44,45^. However, FTIR spectrum of SGO shows a similar pattern to that of GO with the appearance of a new peaks at 1104 cm^−1^ assigned to SO_3_H group which confirm the successful sulfonation of GO to give SGO^46^. Regarding FTIR spectrum of CA, the absorption peaks at 1031 and 1733 cm^−1^ are attributed to C–O, C=O, while the two peaks located at 1367 and 1220 cm^−1^ are ascribed to O=C–OR and C–O–C of the pyranose ring, respectively^47^. In addition, the peaks at 1490, 2940 and 3478 cm^−1^ are assigned to C–H, aliphatic CH_2_ and O–H groups, respectively^48,49^. Finally, the FTIR spectrum of SGO@CA beads shows the main characteristic peaks of SGO and CA asserting the good combination between SGO and CA and the successful fabrication of the SGO@CA beads.

#### XRD

The crystalline structure of the prepared materials was examined with XRD. Figure 2B elucidates the XRD patterns of pristine materials GO, SGO and CA as well as the fabricated SGO@CA beads. XRD profile depicts the discriminative peak of GO at 2θ = 10.25° which confirm the formation of GO from graphite^50^. Meanwhile, the introduction of SO_3_H group to GO during the sulfonation process slightly shifted the characteristic peak of GO from 2θ = 10.25° to 10.60° with obviously decline in the peak intensity, agreeing with Beydaghi et al. study^51^. This result reveals the successful sulfonation of GO to give SGO. The XRD pattern of pure CA points out a peak at 2θ = 8.7° and a broad peak around 2θ = 20°^52^. After the fabrication of SGO@CA beads, a broadening of the main peaks was observed reflecting the successful combination between SGO and CA.

#### TGA

Figure 2C points out the TGA profiles of SGO, CA and SGO@CA beads. All the TGA profiles exhibit a corresponding weight loss to water evaporation at the temperature range of 30–70 °C. The TGA profile of SGO illustrates a weight loss at the temperature range of 70–225 °C, suggesting the decomposition of the unreacted oxygen functional groups. Besides, the pyrolysis of the sulfonated groups took place at the temperature ranges of 225–389 °C^53^. The TGA profile of CA elucidates a spike degradation of CA at the temperature range of 313–390 °C^54^. Interestingly, the TGA profile of SGO@CA beads depicts an ameliorated thermal behavior, granting an advantage to the incorporation of SGO into CA beads.

#### SEM

**Figure 3:**
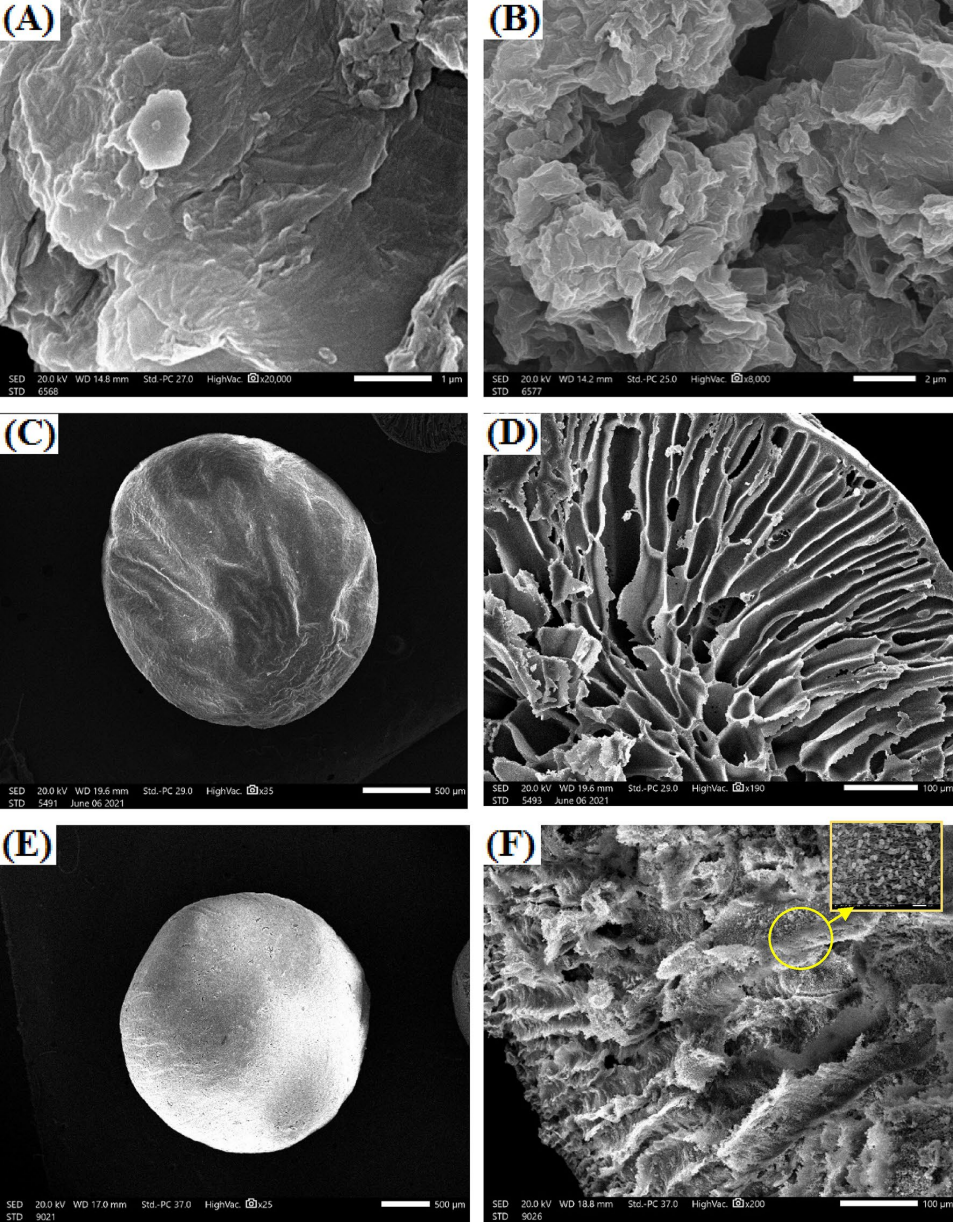
SEM images of (**A**,**B**) SGO, (**C**,**D**) CA beads and (**E**,**F**) SGO@CA composite beads.

The morphologies of the fabricated beads as well as their components were explored using SEM images. SEM images of GO (Fig. 3A) points out the stacked sheets of GO which became more exfoliated and crushed as random piles after the generation of SO_3_H group (Fig. 3B)^55,56^. In addition, SGO sheets are more wrinkled compared to GO sheets, granting SGO the advantage of high surface area^57^. SEM images of pure CA beads (Fig. 3C,D) show the disfigured spherical structure of the outer shell which obviously shrunken, while the cross-sectional structure looks like interconnected macroscale grooves that can act as excellent support for fillers such as SGO that increase the active sites of the beads. Furthermore, SEM image of SGO@CA beads (Fig. 3E) reveals the full moon-like shape of the outer structure with no apparent shrinkage. The cross-section of SGO@CA beads (Fig. 3F) depicts the good distribution of SGO inside the wide grooves of CA which increase its mechanical strength of CA beads as well as their efficiency.

#### XPS

**Figure 4:**
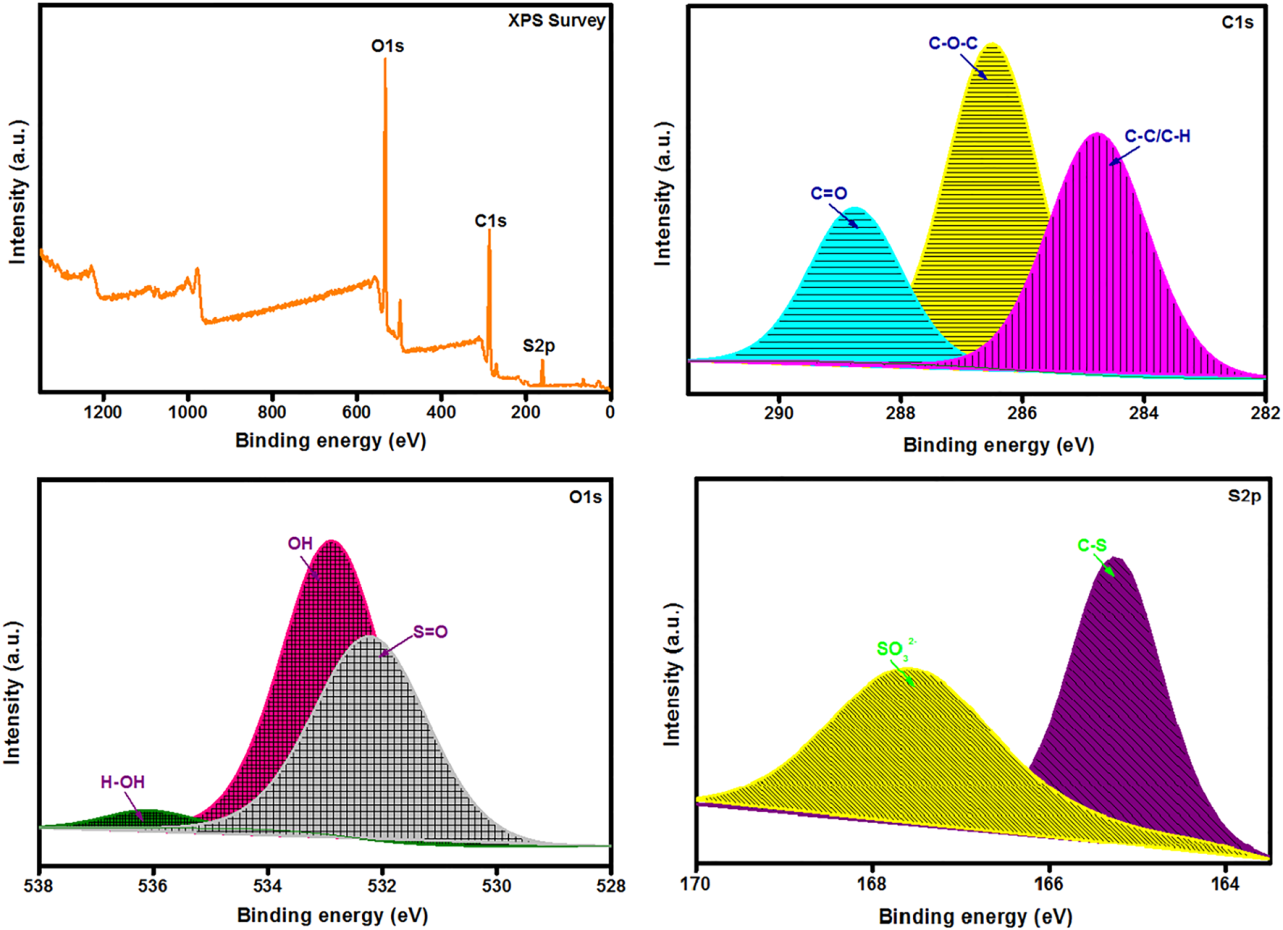
XPS spectra of SGO@CA beads; (**A**) XPS survey, (**B**) C1s, (**C**) O1s and (**D**) S2p.

The elemental composition of the material surfaces could be determined with the aid of XPS analysis. XPS spectrum of SGO@CA beads elucidates the good combination between the pure components since at the peaks at binding energy (BE) of 287.02, 533.37 and 164.78 eV are the relative peaks to C1s, O1s and S2p, respectively as depicted in Fig. 4a. In details, the C1s-XPS spectrum (Fig. 4b) illustrates three peaks at BE of 286.5, 288.75 and 284.76 eV which are ascribed to C–O–C, C=O and C–C/C–H, respectively, which are the main bonding forms in both CA and SGO. Furthermore, the O1s-XPS high resolution spectrum (Fig. 4c) reveals the oxygen-containing functional groups; OH, H–OH and S=O at BE of 532.66, 536.11 and 532.49 eV, respectively. Interestingly, the presence of the peak at 532.49 eV corresponding to S=O confirmed the sulfonation of GO into SGO and further confirms the incorporation of SGO into CA beads. In addition, the S2p-XPS high resolution spectrum (Fig. 4d) pointed out the presence of characteristic peaks of SO_3_^2−^ and C–S at BE of 167.36 and 165.14 eV, respectively. As a result, XPS analysis proved that the fabricated matrix contains many functional groups belonging to both SGO and CA which confirms the successful fabrication of SGO@CA beads.

#### ZP measurements

Surface charge is a crucial point for asserting the role of the electrostatic interaction in the adsorption mechanism, so ZP of CA, SGO and SGO@CA beads were determined (Fig. S1). Data clarified that SGO@CA beads have the highest negative surface charge (− 42.2 mV) while SGO has a zeta potential of − 38.7 mV and that of pure CA was found to be − 35.7 mV at pH 7. This finding may be due to the plenty anionic functional group in SGO backbone (viz., SO_3_H, OH and COOH) that increases the negative charges on the surface of SGO@CA beads. Consequently, SGO@CA beads are assumed to be an excellent candidate for the removal of cationic pollutants like MB.

### Plackett–Burman design

Five factors affecting the MB adsorption capacity were used in the Plackett–Burman design. The adsorption capacity of SGO@CA composite beads varied between 29.6 and 286.1 mg g^−1^ during testing, as revealed in Table S2. Time, pH, adsorbent dosage and dye concentration all had a beneficial effect on dye adsorption capacity; however, temperature had a negative effect. The following polynomial model describes the relationship between the five components and sorption capacity:

5 $$Y=103.27+1.525{X}_{1}-2.718{X}_{2}-3930.83{X}_{3}-0.352{X}_{4}+0.557{X}_{5}$$

As displayed in Table 1, ANOVA test was used to estimate the variance, and the results show that the variables have a statistically significant relationship. In addition, the model's R-squared and adjusted R-squared values indicate that it is the most fit. Time, dye concentration, dose, and pH were chosen for further optimization based on the expected coefficients, t values, and P values since these variables had the most noteworthy responses on the adsorption capacity and R-squared = 0.92. All trials were run using the (− 1) level of the parameter variable that had a negative significant influence on subsequent optimization experiments.

**Table 1:** Analysis of variance (ANOVA) for Plackett–Burman experiments for dye adsorption by SGO@CA composite beads.

Source	Degree of freedom	Sum of squares	Mean square	F ratio	P value
Regression	5	83,509.2	16,701.8	15.8	0.002
Residual	6	6306.9	1051.1		
Total	11	89,816.2			

#### Box–Behnken design

**Figure 5:**
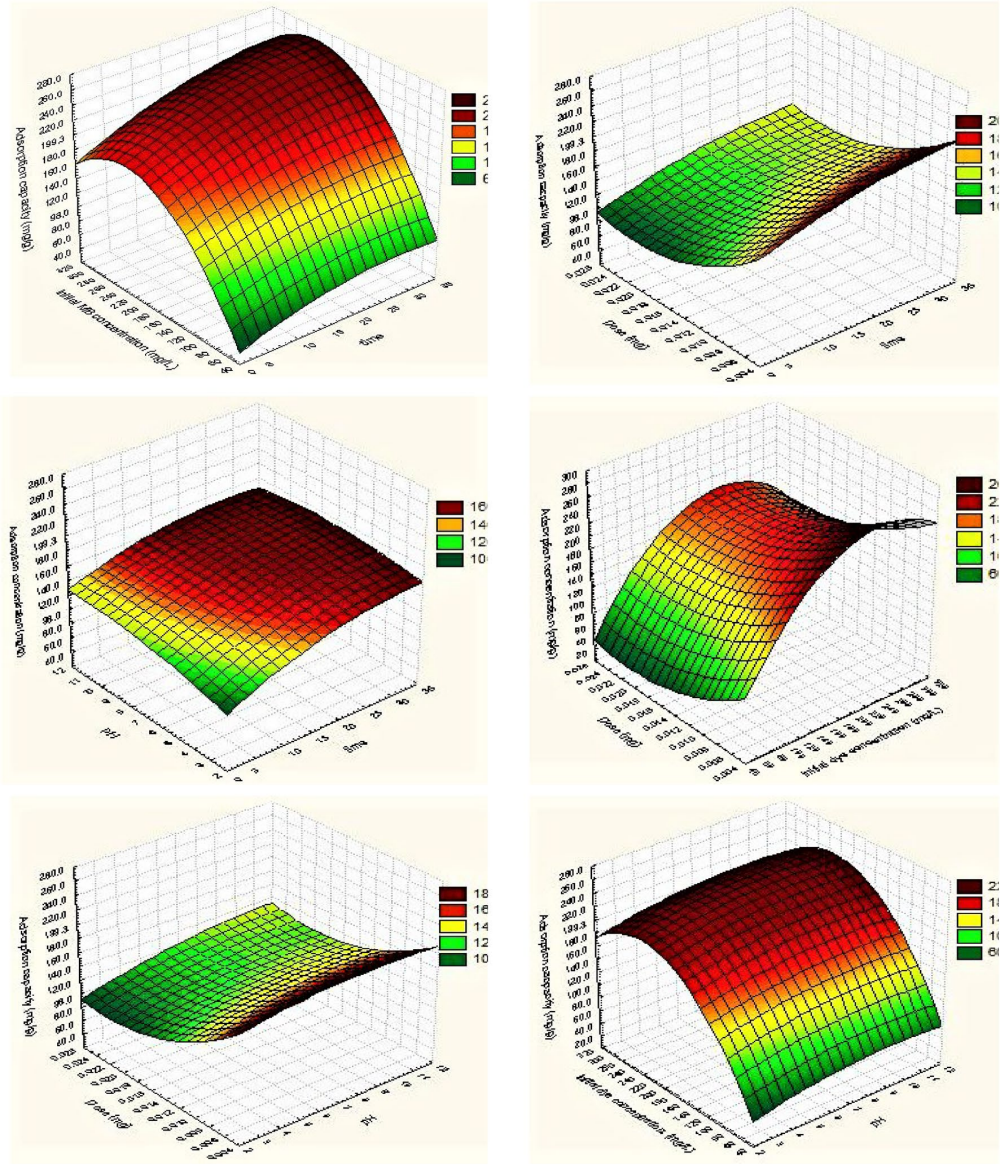
Three-dimensional response surface plots of MB adsorption capacity using SGO@CA composite beads.

Twenty-seven trials were used to complete the response surface methodology (RSM) utilizing Box–Behnken design based on results from the Plackett–Burman design for the four most important factors impacting dye adsorption capacity at three levels. A linear multiple regression analysis was used to explore the data. Multiple correlation coefficients R and the determination coefficient R^2^ are used to evaluate the regression equation at the model level^58^. The closer R^2^ is to 1.0, the higher the correlation between the measured and predicted values is. R^2^ was found to be 0.995 in this study. The R^2^ value of 0.99 is a measure of the model's ability to predict. Each of the four independent variables is depicted in the multiple linear regression models. As exposed in Table 2, the produced adsorbents successfully removed dye using an ANOVA test. There is strong evidence to support the model's statistical significance, as shown by its high F-ratio and incredibly low probability P value^59,60^ (P value 0.01). Using Statistica 7.0 software, three-dimensional plots were created to examine the influence of various variables on the adsorption capacity (Fig. 5). For example, as time and dye concentration increased, the adsorption capacity of SGO@CA composite beads increased, and this was achieved with both low and moderate pH values as well as low adsorbent doses. Second-order polynomial functions adapted to laboratory data were used to find the best location (linear optimization algorithm).

**Table 2:** Analysis of variance (ANOVA) for Box–Behnken experiments for MB adsorption by SGO@CA composite beads.

Source	Degree of freedom	Sum of squares	Mean square	F ratio	P value
Regression	14	74,005.6	5286.1	89.4	6.42E−10
Residual	12	708.8	59.0		
Total	26	74,714.5

6 $$Y=152.13+20.94 {X}_{1}+8.82 {X}_{2}-32.71 {X}_{3}+66.83{ X}_{4}-1.97 {X}_{1}{X}_{2}-0.921 {X}_{1}{X}_{3}+10.73 {X}_{1}{X}_{4}+6.72 {X}_{2}{X}_{3}-2.52 {X}_{2}{X}_{4}- 1.9 {X}_{3}{X}_{4}+1.5 {{X}_{1}}^{2}-7.24 {{X}_{2}}^{2}+1.76 {{X}_{3}}^{2}- 6.47{{X}_{4}}^{2}$$

It was found that 30 min of exposure duration, 300 g L^−1^ of MB concentration, pH 6 and 0.005 g of adsorbent dose yielded the highest adsorption capacity when the polynomial model's maximum point was taken into consideration.

The improvement in the adsorption process with the increase of the pH values **(**Fig. 5) can be attributed the decrease in H^+^ ions concentration in the solution that competes with MB for the binding sites of the beads^61^. In addition, the existence of the abundant negative charges on the 30%SGO@CA surface strengthens the attraction forces between MB and the beads, reflecting that the electrostatic interaction mainly controls the MB adsorption mechanism^50^.

On the other hand, increasing the adsorbent dose from 0.005 to 0.025 g significantly decreased the adsorption capacity this can be explained increasing the aggregation tendency of adsorbent particles, and consequently, the surface area directly dwindles^62^. Consequently, the number of the vacant active sites at a fixed MB dye concentration increases, resulting in a decline in the adsorption capacity value. Conversely, increasing the adsorbent dose increases the removal (%) from 76.07 to 94.94%, which can be described by presence of a plethora of adsorption sites onto the 30%SGO@CA surface^63^.

#### Verification of the model

Verification experiments were conducted to determine the quadratic polynomial's accuracy under the expected ideal conditions. Following the formula, the percentage of accuracy was determined according to the following equation:

7 $$Accuracy=\frac{{Y}_{Experiment}}{{Y}_{Calculated}} \times 100$$

Experimentally, an adsorption capacity of 277.45 mg g^−1^ was achieved, implying that the model accuracy calculated was 97.89%.

### Impact of SGO proportion

**Figure 6:**
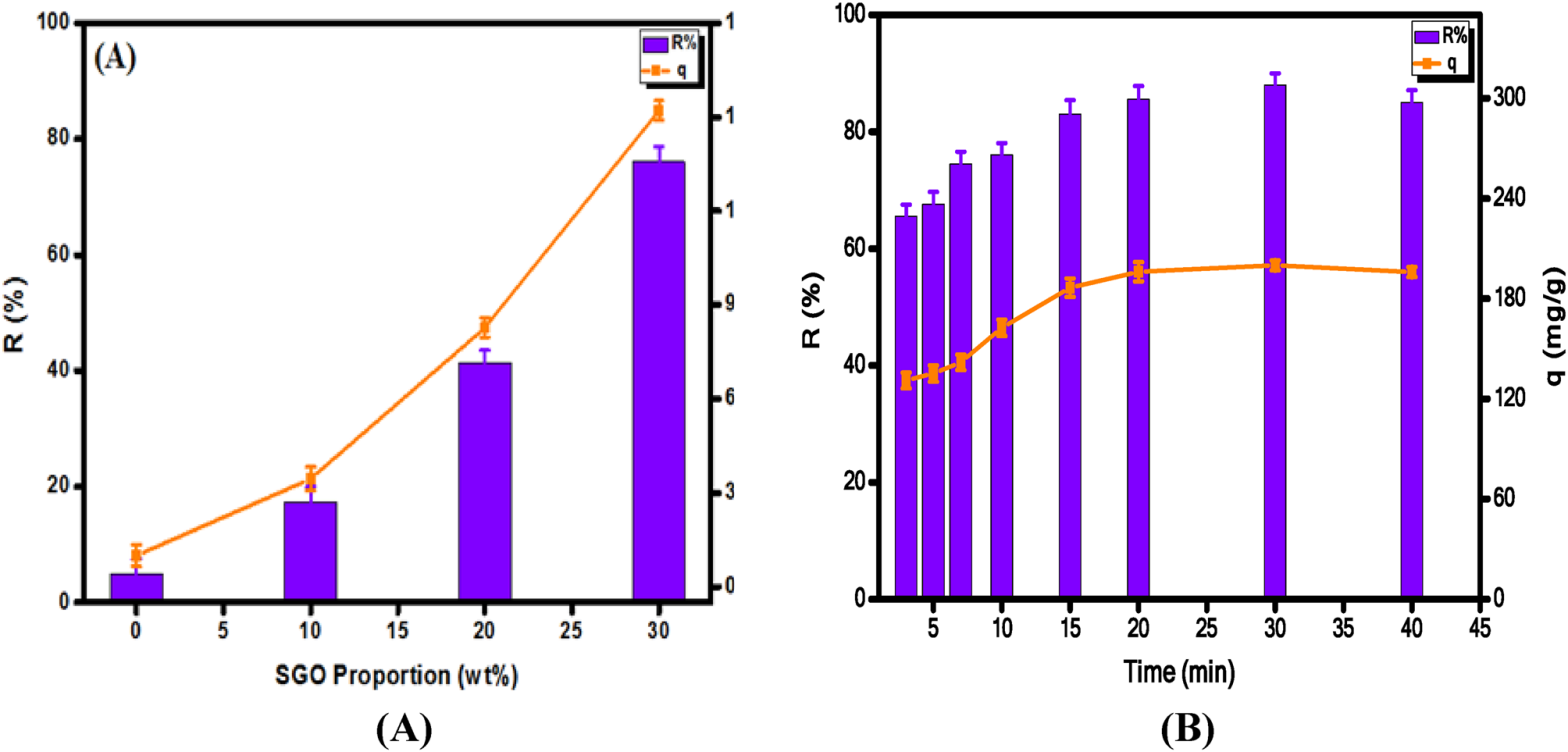
(**A**) Effect of SGO and (**B**) effect of contact time on the adsorption of MB dye.

Figure 6A points out the inferior adsorption performance of CA beads toward MB since the removal percent and the adsorption capacity of MB were 5.00% and 10.00 mg g^−1^, respectively. Furthermore, it was noticed an incredible amelioration in the removal percent of MB from 17.24 to 76.07% and the adsorption capacity from 34.48 to 152.14 mg g^−1^ with raising the incorporated amount of SGO into CA beads from 10 to 30% wt, respectively. This finding is most likely due to the existence of plenty of anionic binding groups in SGO (viz., OH, COOH and SO_3_H) that strongly attract the cationic MB molecules from their bulk solution through the electrostatic interaction process. In addition, SGO boosts the surface area of CA beads and the adsorption properties of the developed composite beads enhanced accordingly. Thus, beads containing 30% SGO were opted for the subsequent adsorption experiments.

### Impact of time and adsorption kinetics

Figure 6B, denoted the consequence of contact time on the quantity of adsorbed MB dye by the formulated floated beads. It was noticed that the adsorption capacity of MB dye increased hastily in the first adsorption stage. Therefore, only 3 min were enough for adsorbent beads to reach 131 mg g^−1^ capacity with a removal efficiency of 65%. Subsequently, a slow adsorption rate was observed with increasing time until reached the equilibrium within 30 min (88%R, ~ 200 mg g^−1^). The rapid increment in the adsorption capacity and the removal (%) values at first stage could be attributed to the existence of adequate free adsorption sites on the surface of adsorbent beads. This directly causes an increase in the concentration gradient between adsorbate in solution and adsorbate on the adsorbent surface. The number of MB molecules that diffuse through the film fluid around adsorbent beads increases and the adsorption rate at initial phase increases accordingly. Over the time, increasing the contact time beyond 30 min there was no significant consequence on the adsorption rate, since all adsorption sites were saturated within 30 min.

**Figure 7:**
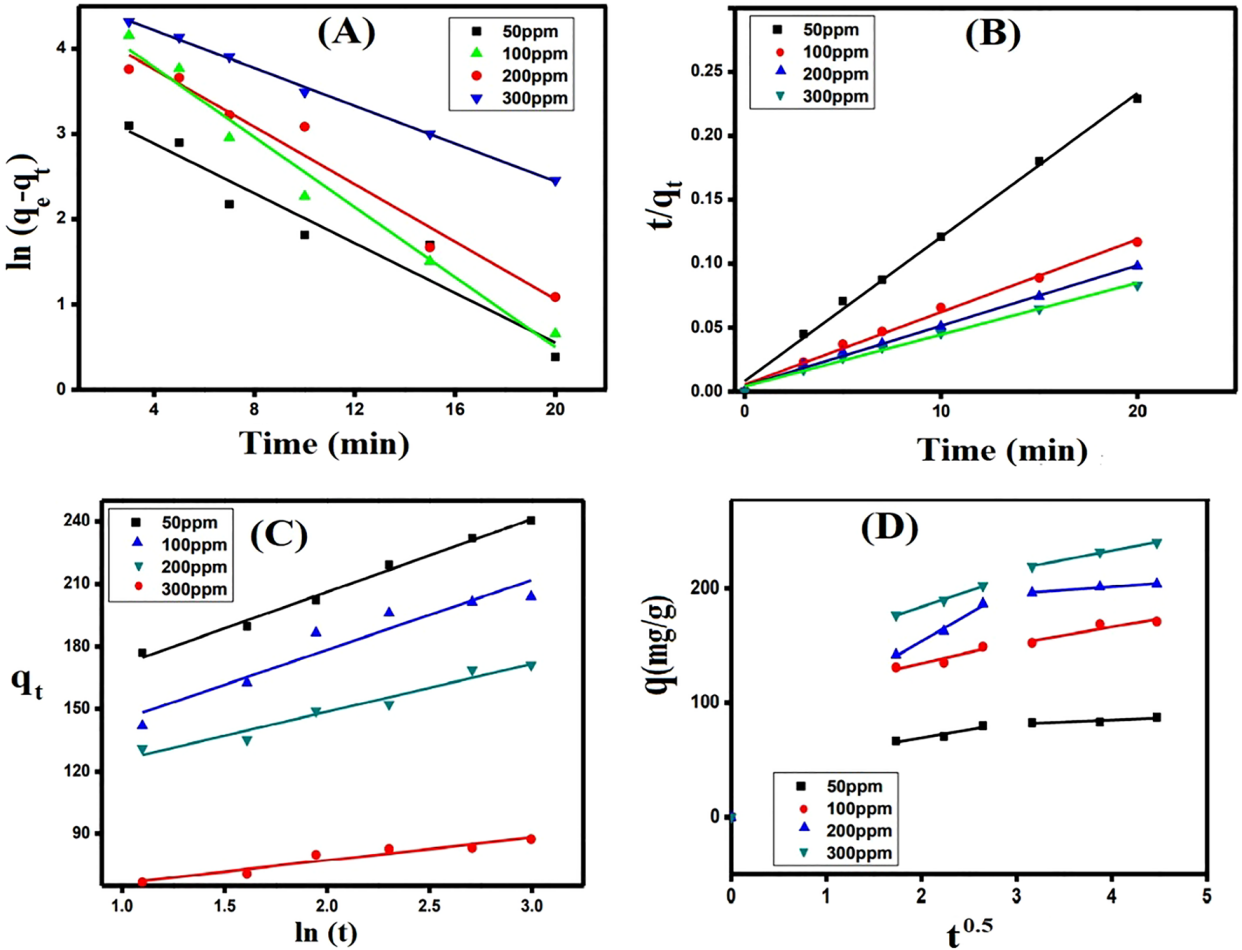
(**A**) The Pseudo-first order, (**B**) the Pseudo-second order, (**C**) Elovich and (**D**) intra-particle diffusion kinetic models for the adsorption of MB dye onto SGO@CA composite beads.

The experimental data were examined using the Pseudo-first order, Pseudo-second order, Elovich and Intra-particle diffusion kinetic models (Fig. 7A–D) to explain the adsorption mechanism of MB dye onto SGO@CA composite beads^64^. The following equations symbolize the linear forms of the studied kinetic models^65^.

8 $$\mathrm{Pseudo}-\mathrm{first}-\mathrm{order:}\mathrm{ ln}({\mathrm{q}}_{\mathrm{e}}-{\mathrm{q}}_{\mathrm{t}})={\mathrm{lnq}}_{\mathrm{e}}-{\mathrm{k}}_{1 }\mathrm{t} $$

9 $$\mathrm{Pseudo}-\mathrm{second}-\mathrm{ order:} \mathrm{t}/{\mathrm{q}}_{\mathrm{t}}=1/{\mathrm{k}}_{2}{\mathrm{q}}_{\mathrm{e}}^{2}+ 1/{\mathrm{q}}_{\mathrm{e}}\mathrm{t}$$

10 $$\mathrm{Elovich\, model:} {\mathrm{q}}_{\mathrm{t}}=1/\mathrm{\beta ln }(\mathrm{\alpha \beta })+ \frac{1}{\upbeta }\mathrm{ln} t$$

11 $$\mathrm{Intra}-\mathrm{particle\, diffusion\, model:} {\mathrm{q}}_{\mathrm{t}}={\mathrm{K}}_{\mathrm{P }}{\mathrm{t}}^{0.5}+\mathrm{C}$$

where q_e_ and q_t_ represent the amount of adsorbed MB dye onto SGO@CA composite beads at equilibrium and at time t, respectively. k_1_ is the rate constant of the pseudo-first order and k_2_ signifies the rate constant of the pseudo-second order. α and β denote Elovich coefficients. K_p_ is the intra-particle diffusion rate constant (mg g^−1^ min^−1^), while C refers to the intercept.

The results (Table 3) indicated that the best kinetic model to describe the adsorption process of MB dye onto SGO@CA composite beads is the Pseudo-second order, which was proved also from the similarity between the computed q values from the Pseudo-second order and the actual experimental values. Additionally, Elovich model clarified that the rate of adsorption was higher than desorption, since α values were higher than β values. These increments of α values advowson the fast adsorption of MB dye onto SGO@CA composite beads within 30 min.

**Table 3:** Data of Pseudo-first order, Pseudo-second order and Elovich kinetic models for the MB dye adsorption onto SGO@CA composite beads.

Kinetic models and parameters	Concentration (mg L^−1^)			
	50	100	200	300
q_e,__exp_ (mg g^−1^)	82.7	152.1	196.1	219.2
**Pseudo 1st order**				
q_e,cal_ (mg g^−1^)	47.9	134.5	164.9	164.7
k_1_ (min^−1^)	0.19986	0.25196	0.29013	0.20565
R^2^	0.807	0.916	0.981	0.941
**Pseudo 2nd order**				
q_e,cal_ (mg g^−1^)	88.81	176.05	211.86	246.91
k_2_ (g mg^−1^ min^−1^)	0.0153	0.006	0.0052	0.0040
R^2^	0.995	0.992	0.994	0.992
**Elovich**				
α (mg g^−1^ min^−1^)	1713.5	2003	928.3	1719
β (g mg^−1^)	0.09132	0.04351	0.0298	0.0286
R^2^	0.904	0.946	0.891	0.989

As shown from data obtained from Intra-particle diffusion kinetic model (Table S6), the adsorption of MB dye onto SGO@CA composite beads carried out within two steps. The first step concerned was the diffusion of dye molecules from the bulk solution to the external surface of MB dye onto SGO@CA composite beads. The second step that involves the diffusion of MB dye molecules to the pores of SGO@CA composite beads. It is noted that values of C are not equal to zero at all the initial MB dye concentrations which revealed that the intra-particle diffusion is not the only rate-controlling step^24^. Besides, the K_p_ values increased with increasing the initial dye concentration, indicating that the adsorption of MB dye onto SGO@CA composite beads is favorable at high concentration and supports improved rate of adsorption.

### Impact of initial dye concentration and isotherms studies

**Figure 8:**
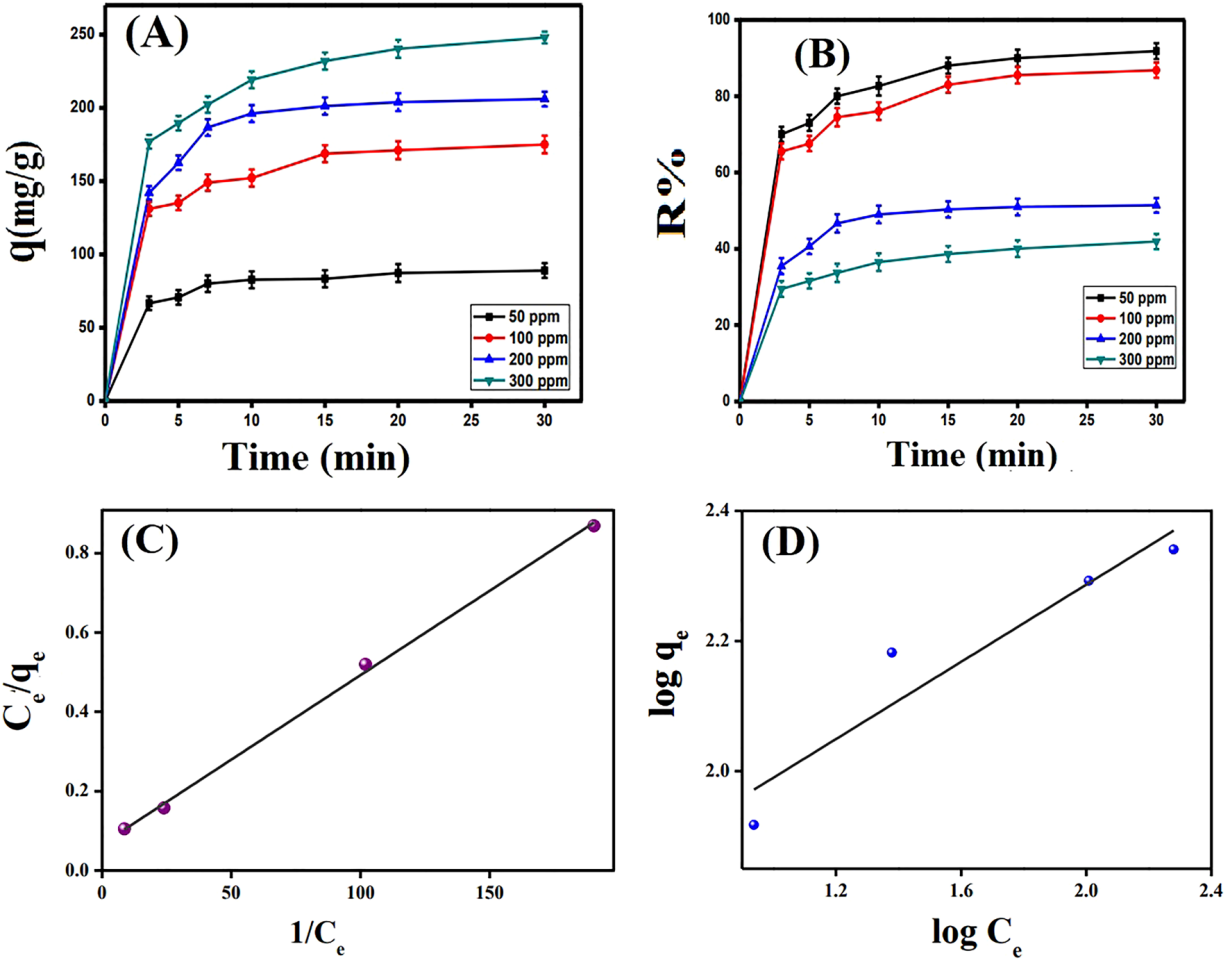
Impact of initial MB concentration on (**A**) adsorption capacity and (**B**) removal (%). (**C**) The linear isotherm model of Langmuir and (**D**) Freundlich model for the MB dye adsorption onto SGO@CA composite beads.

Indeed, the increment in the contaminant concentration renders its driving force stronger than the mass transfer resistance. So, the migration of contaminant species from their bulk solution to the surface of the adsorbent increases^66^. In light of this fact, the increase in the MB initial concentration from 50 to 300 mg L^−1^ ameliorates the adsorption capacity of MB onto 30%SGO@CA from 87.33 to 240.34 mg g^−1^ (Fig. 8A). Nonetheless, this raising in the MB initial concentration declines the removal percent from 90.00 to 40.06% (Fig. 8B) at which the adsorption sites are inadequate for the excess amounts of MB molecules.

The adsorption efficacy of SGO@CA composite beads in addition to the adsorbent-adsorbate interactions were investigated using adsorption isotherm models. The linear forms of Langmuir, Freundlich, Temkin and Dubinin–Radushkevich (D–R) isotherm models were applied according to the following equations^19,65^:

12 $$\mathrm{Langmuir\, equation:} \frac{{\mathrm{C}}_{\mathrm{e}}}{{\mathrm{q}}_{\mathrm{e}}}=\frac{1}{{\mathrm{K}}_{\mathrm{L}} {\mathrm{q}}_{\mathrm{m}}}+\frac{{\mathrm{C}}_{\mathrm{e}}}{{\mathrm{q}}_{\mathrm{m}}}$$

13 $$\mathrm{Freundlich\, equation:} \mathrm{ log}{\mathrm{q}}_{\mathrm{e}}={\mathrm{logK}}_{\mathrm{F}}+\frac{1}{\mathrm{n}}{\mathrm{logC}}_{\mathrm{e}}$$

14 $$\mathrm{Temkin\, equation:} {\mathrm{q}}_{\mathrm{e}}=\mathrm{B lnA}+{\mathrm{BlnC}}_{\mathrm{e}}$$

15 $$\mathrm{B}=RT/b$$

16 $$\mathrm{D}-\mathrm{R equation:} \mathrm{ln}{\mathrm{q}}_{\mathrm{e}}=\mathrm{ln }{\mathrm{q}}_{\mathrm{s}}-{\mathrm{K}}_{\mathrm{ad }}{\upvarepsilon }^{2}$$

17 $$\upvarepsilon =\mathrm{RT ln }(1+1/{\mathrm{C}}_{\mathrm{e}} )$$

18 $${\mathrm{R}}_{\mathrm{L}}=1/(1+b {C}_{0} )$$

where q_e_, q_m_ and C_e_ are the equilibrium adsorption capacity, the monolayer adsorption capacity, and the remaining concentraion of MB dye at equilibrium, respectively. K_L_ represents Langmuir constant, while K_F_ and n signify the Freundlich constants, respectively. B and A are the Temkin constant and the equilibrium binding constant, respectively. R and T are the gas constant (8.314 J mol^−1^ k^−1^) and the absolute temperature. ε represents the Polanyi potential (kJ mol^−1^), while K_ad_ is a constant related to mean free energy of adsorption per mole of adsorbate (mol^2^ kJ^−2)^. q_s_ is the saturation adsorption capacity (mg g^−1^), while R_L_ signifies the dimensionless separation factor.

The results depicted in Table 4, Fig. 8C,D and Fig. S2 signified that the adsorption of MB dye onto SGO@CA composite beads obeyed Langmuir (R^2^ = 0.998) and Temkin (R^2^ = 0.945) models compared to Freundlich (R^2^ = 0.858) and D–R (R^2^ = 0.926) models. From Langmuir model; the maximum adsorption capacity of MB dye onto SGO@CA composite beads is 234.74 mg g^−1^ which is in a good agreement with the experimentally determined value (219.2 mg g^−1^). Furthermore, the dimensionless separation factor (R_L_) computed values range from 0 to 1, demonstrating that the interaction between SGO@CA composite beads and MB dye is favorable at both low and high initial MB concentrations^63^. Otherwise, the calculated Freundlich constant (n = 3.37) indicated the preference for this uptake process. According to Temkin model (Fig. S2) MB dye adsorption process occurs through physical adsorption of MB dye onto SGO@CA composite beads, with a sorption value of less than 1.0 kcal mol^−1^ this result is agreed with D–R model, since the bonding energy is 0.2 kJ mol^−1^ which was less than 8 kJ mol^−1^. Generally, physical adsorption occurs via weak van der Waals interactions, hence the uptake of E = 1/√2K_ad_ needs low adsorption energy^67^.

**Table 4:** The parameters derived from isotherm models for the MB dye adsorption onto SGO@CA composite beads.

Langmuir model			Freundlich model			
q_m_ (mg g^−1^)	b (L mg^−1^)	R^2^	K_F_	n	R^2^	
234.74	0.0617	0.998	49.35	3.37	0.858	

Temkin				D–R		
A (L g^−1^)	B (J mol^−1^)	b (KJ mol^−1^)	R^2^	q_s_	K_ad_ (mol^2^ kJ^−2^)	R^2^
1.05	42.2	0.058	0.945	196.7	1.2*10^-5	0.926

### Impact of temperature and thermodynamics studies

**Figure 9:**
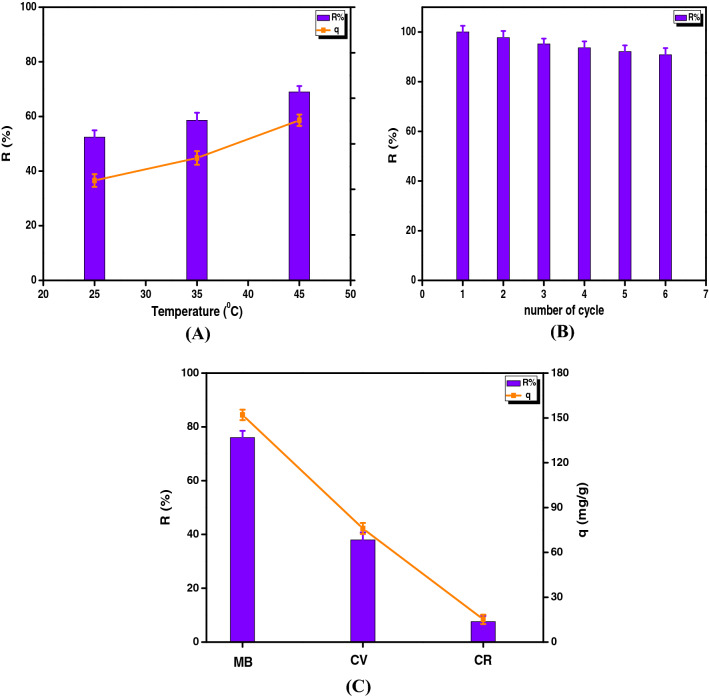
(**A**) Effect of adsorption temperature on the adsorption process, (**B**) reusability and (**C**) selectivity of SGO@CA composite beads toward cationic and anionic dyes.

As shown in Fig. 9A, increasing the temperature from 25 to 45 °C resulted in a noticeable enhancement in both removal percent and adsorption capacity from 52.4% and 104 mg g^−1^ to 68% and 137 mg g^−1^, respectively. These results signified that the adsorption of MB dye onto SGO@CA composite beads is an endothermic process where raising the temperature assists the MB dye to move easily towards the SGO@CA composite beads. Increasing adsorption temperature improves the diffusion rate of the MB molecules through the external boundary layer and internal pores of the adsorbent, causing an increase in the attached amount of MB on the SGO@CA composite surface. These results agreed with other reported studies^22^.

Fundamental thermodynamic parameters (ΔH°, ΔS°, ΔG°) were determined to realize the nature and the mechanism of MB dye adsorption onto SGO@CA composite beads. Changes in entropy (ΔS°), change in enthalpy (ΔH°) and change in free energy (ΔG°) were calculated according to the following equations^50,63^:

19 $${K}_{e}=\frac{{C}_{Ae}}{{C}_{e}}$$

20 $${\mathrm{lnK}}_{\mathrm{e}}=\frac{\Delta {\mathrm{S}}^{\mathrm{o}}}{\mathrm{R}}-\frac{\Delta {\mathrm{H}}^{\mathrm{o}}}{\mathrm{RT}}$$

21 $${\Delta \mathrm{G}}^{\mathrm{o}}={\Delta \mathrm{H}}^{\mathrm{o}}-\mathrm{T}{\Delta \mathrm{S}}^{\mathrm{o}}$$

where K_e_ represents the thermodynamic equilibrium constant, while C_e_ and C_Ae_ are the concentration of MB dye in the bulk solution and onto the surface of SGO@CA beads at equilibrium, respectively. R and T refer to the gas constant (8.314 J mol^−1^ K^−1^) and the medium temperature (K), respectively.

These parameters were evaluated via running the adsorption of MB dye on SGO@CA composite beads at different temperatures. The endothermic character of the process is evidenced by the increase in Kc values as temperature rises, as well as the positive ΔH° value. As shown in Table S7, the magnitude of ΔH° is highly influenced by the types of forces involved in the adsorption process, with the obtained ΔH° value (+ 3.059 kJ mol^−1^) that indicated the presence of van der Waals forces, dipole bond forces, hydrogen bonding, and/or coordination exchange in this study. Furthermore, the entropy change (ΔS°) was positive (+ 94.72 J mol^−1^ K^−1^), indicating that there was an increase in the randomness at the adsorbent–adsorbate interface during the MB dye adsorption. In addition, as the temperature rises, the negative values of ΔG° decreases, indicating that the process becomes more feasible and spontaneous.

### Reusability and selectivity of adsorbent

From an economical point of view, the ability of an adsorbent to be regenerated and reused is critical for lowering production costs. SGO@CA composite beads had outstanding adsorption properties, as demonstrated in Fig. 9B. The results refereed that the removal (%) of SGO@CA composite beads still more than 90% after six consecutive adsorption–desorption cycles. This reflects its high stability and activity that gained from the combination of SGO and CA as beads. Besides, the SGO@CA composite beads floating nature and bead shape facilitate separation and reuse. To further identify the universality of selective adsorption, further cationic crystal violet (CV) and anionic Congo red (CR) dyes were involved in the adsorption medium with cationic MB dye to assess the selective adsorption characteristic of SGO@CA composite beads as depicted in Fig. 9C. The results signified that the developed adsorbent showed better affinity towards adsorption of cationic dyes (MB and CV) compared to the anionic CR dye. In addition, the developed adsorbent beads were more selective to MB dye rather than CV dye, which recorded maximum removal (%) of 76% and adsorption capacity of 152 mg g^−1^ compared to 38% and 76 mg g^−1^. On the other hand, the anionic CR dye recorded the lowest values of 7.6% and 15.2 mg g^−1^ which mainly attributed to the repulsive forces between its negative charges and the negatively charged adsorbent surface.

### The possible adsorption mechanism of MB onto SGO@CA beads

**Figure 10:**
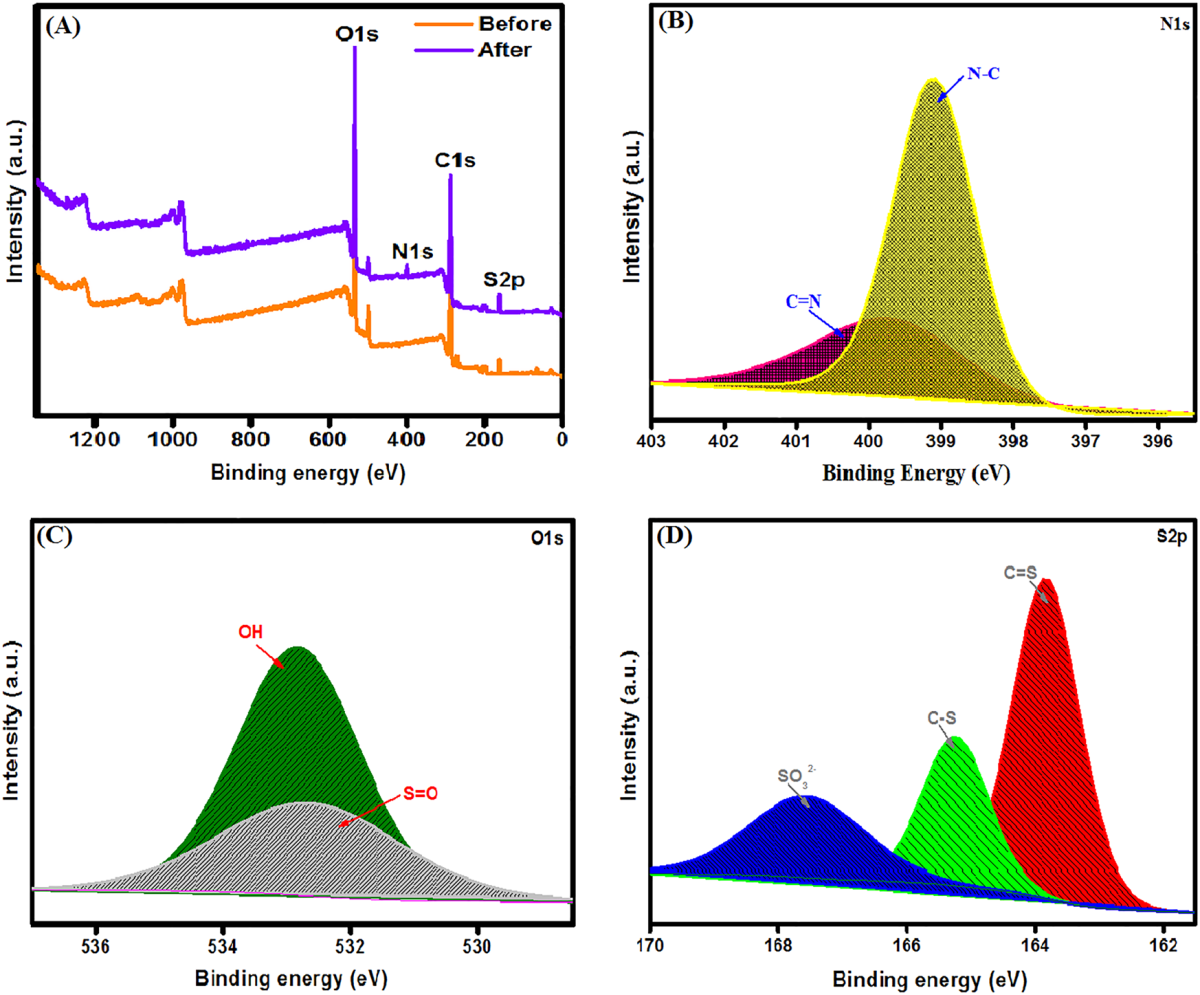
XPS spectra of MB-loaded SGO@CA; (**A**) XPS survey, (**B**) N1s, (**C**) S2p and (**D**) O1s.

The XPS wide spectrum of SGO@CA (Fig. 10) after the MB adsorption shows the belonging peaks to N1s at BE of 400.17 eV. Besides, the S2p peak shifted from 164.78 to 165.08 eV with an increase in the peak intensity. These observations assert the adsorption of MB dye onto SGO@CA beads. The N1s-XPS spectrum points out the N-containing functional groups; N–C and N=C at BE of 399.10 and 399.72 eV, respectively, which is one more proof to confirm the MB adsorption onto the beads. Several studies reported the impossibility to the large MB molecules (length = 13.82 or 14.47 A^°^ and width = 9.5 A^°^) to completely enter the porous of adsorbents, suggesting the partial entering of MB molecules via the interaction between the Lewis acid SO_3_^2−^ and the Lewis base N(CH_3_)_2_ and C_6_H_5_^68^. Moreover, the O1s-XPS spectrum depicts the peaks shift from 532.66 and 532.49 to 532.83 and 532.64 eV, respectively. In addition, the peak shift in the S2p-XPS spectrum from 165.13 and 167.14 to 165.24 and 167.56 eV, respectively, reflects the electrostatic interaction between MB molecules and SGO@CA beads. Also, ZP measurements confirm this finding since SGO@CA was highly negatively charged (− 42.2 mV) at a neutral medium which facilitates the attraction of the cationic MB from their bulk solution. Furthermore, the appearance of the belonging peak to S=O at BE of 163.83 eV, confirming the adsorption of MB onto the beads. Additionally, van der Waals force, π–π interaction, hydrogen bond and n–π interaction contribute to the adsorption mechanism of MB onto SGO@CA beads^64,69^.

## Conclusion

This study reported the fabrication of reusable, easy separable adsorbent based on sulfonated graphene oxide incorporated cellulose acetate floated beads for fast adsorptive removal of cationic MB dye. The developed SGO@CA composite beads were characterized by various analysis tools. The results signified that the surface of SGO@CA beads exposed highly negative charges reached − 42.2 mV. The RSM was used to systematically determine the ideal amounts of the four most effective Plackett–Burman Factorial Design variables. Experimental and predicted results had a strong connection, according to the quadratic model used in this study. In addition, the second-order regression model has been properly adjusted to the experimental data by using an ANOVA analysis that has an R^2^ value ∼ 1. Further, the best optimized values derived from the maximum point of the polynomial model were attained. The results clarified also that the adsorption equilibrium was attained quickly within 30 min, while the adsorption capacity was noticeably improved from 34.48 to 152.14 mg g^−1^ with increasing SGO content from 10 to 30wt%. The experimental data followed the pseudo-second order kinetic model, while data agreed with Langmuir isotherm model with a maximum adsorption capacity of 239.8 mg g^−1^. The thermodynamic studies implied that the process was endothermic and spontaneous. Besides, the reusability study also reveals that SGO@CA composite beads still retains respectable adsorption properties for six consecutive cycles. The appealing characteristics including fast adsorption, facile separation, better reusability and higher adsorption capacity strongly suggest the applicability of the formulated SGO@CA floated beads for adsorptive removal of toxic cationic dyes from their aquatic bodies.

## Supplementary Information

Supplementary Information.

## Data Availability

The data presented in this study are available on request from the corresponding author.
