# Supplementary material for: Sulfonated graphene oxide impregnated cellulose acetate floated beads for adsorption of methylene blue dye: optimization using response surface methodology
Source: Sci Rep. 2022 Jun 4;12:9339. doi: 10.1038/s41598-022-13105-4 (PMC9167308; doi:10.1038/s41598-022-13105-4)
Supplement: Supplementary file 1 — Supplementary Information. [file 41598_2022_13105_MOESM1_ESM.docx]

**Supplementary data**


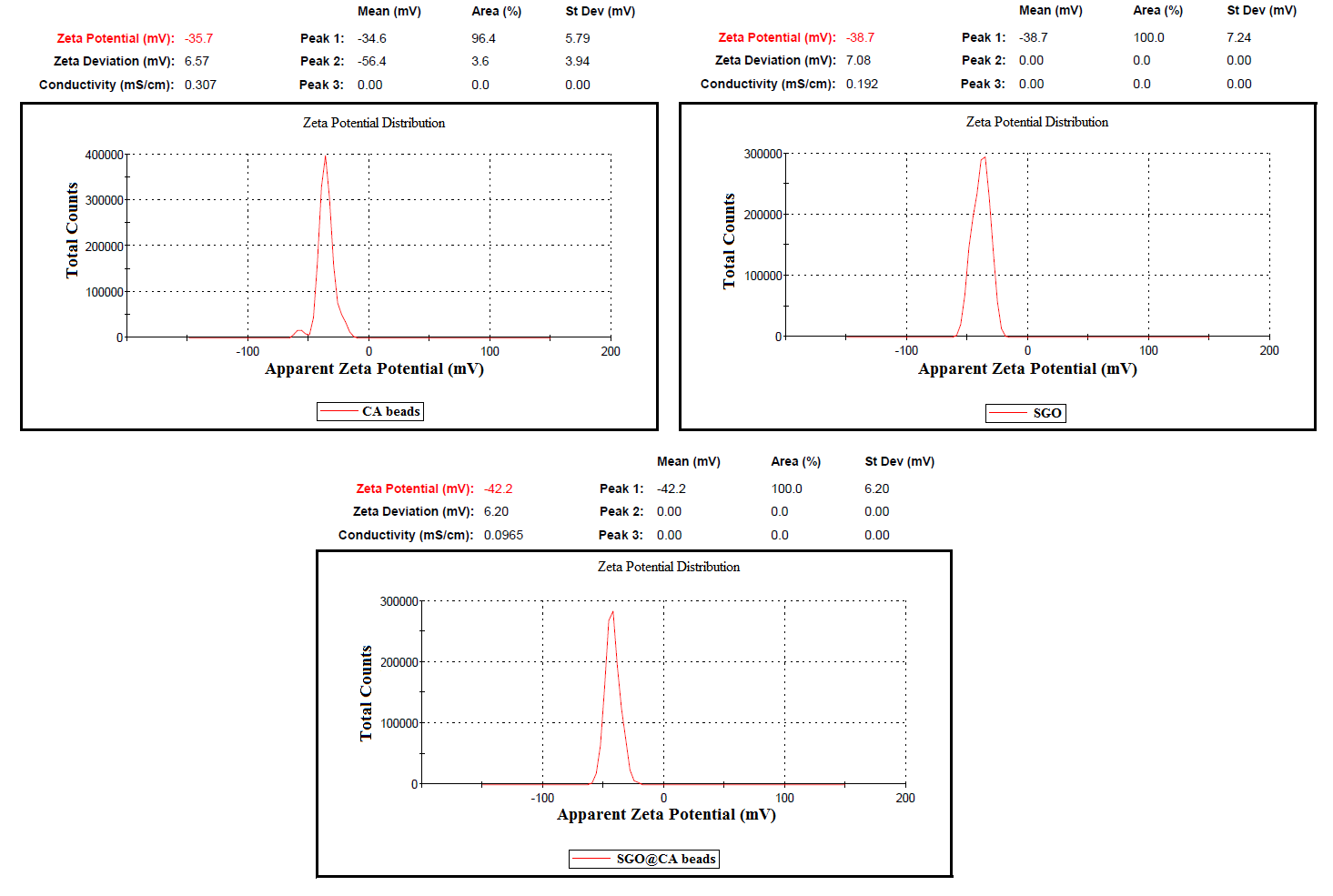


**Fig. S1.** ZP measurements of CA beads, SGO and SGO@CA composite beads.

| **(A)** | **(B)** |
| --- | --- |

**Fig. S2.** The linear isotherm model of **(A)** Temkin and **(b)** D-R for the MB dye adsorption onto SGO@CA composite beads.

**Table S1:** Variables affecting MB adsorption capacity process and tested levels for Plackett-Burman experiment.

| **Variable** | **Variable code** | **Low level (-1)** | **High level (+1)** |
| --- | --- | --- | --- |
| Time (min) | X1 | 10 | 30 |
| pH | X2 | 3 | 11 |
| Adsorbent dose (g) | X3 | 0.005 | 0.025 |
| Temperature (^o^C) | X4 | 25 | 55 |
| Initial dye concentration (g/L) | X5 | 50 | 300 |

**Table S2:** Randomized Plackett-Burman experimental design for evaluating factors affecting dye adsorption capacity using SGO@CA composite beads.

| **Trial** | **Time** | **pH** | **Adsorbent dose (g)** | **Temperature (**^o^C) | **Initial dye concentration (mg/L)** | **Adsorption capacity (mg/g)** |
| --- | --- | --- | --- | --- | --- | --- |
| **1** | 1 | 1 | 1 | 1 | -1 | 46.6 |
| **2** | -1 | 1 | -1 | 1 | -1 | 29.6 |
| **3** | 1 | -1 | -1 | -1 | -1 | 117.4 |
| **4** | 1 | -1 | 1 | -1 | -1 | 55.9 |
| **5** | 1 | 1 | -1 | -1 | 1 | 286.1 |
| **6** | -1 | -1 | 1 | 1 | -1 | 49.8 |
| **7** | -1 | 1 | 1 | -1 | 1 | 143.3 |
| **8** | -1 | -1 | 1 | -1 | 1 | 136 |
| **9** | -1 | 1 | -1 | -1 | -1 | 96.6 |
| **10** | 1 | 1 | 1 | 1 | 1 | 146.7 |
| **11** | -1 | -1 | -1 | 1 | 1 | 235.3 |
| **12** | 1 | -1 | -1 | 1 | 1 | 285 |

**Table S3:** The levels of variables chosen for the Box–Behnken optimization experiments for the adsorption of MB dye by SGO@CA composite beads.

| **Variable** | **Variable code** | **-1** | **0** | **+1** |
| --- | --- | --- | --- | --- |
| Time (min) | X1 | 3 | 10 | 30 |
| pH | X2 | 3 | 7 | 11 |
| Adsorbent dose | X3 | 0.005 | 0.01 | 0.025 |
| Initial dye concentration (mg/L) | X4 | 50 | 100 | 300 |

**Table S4:** Box–Behnken factorial experimental design, representing the response of dye adsorption capacity (mg/g) as influenced by time, pH, adsorbent dose and initial MB concentration for SGO@CA composite beads.

| **Trial** | **X_1_** | **X_2_** | **X_3_** | **X_4_** | **Adsorption capacity (mg/g)** | |  |
| --- | --- | --- | --- | --- | --- | --- | --- |
|  |  |  |  |  | **Measured** | **Predicted** | **Accuracy (%)** |
| **1** | +1 | 0 | 0 | -1 | 240.3 | 245.7 | 97.8 |
| **2** | 0 | 0 | +1 | -1 | 55.9 | 49.7 | 112.3 |
| **3** | 0 | +1 | +1 | 0 | 125.6 | 129.4 | 96.9 |
| **4** | 0 | +1 | -1 | 0 | 173 | 181.4 | 95.3 |
| **5** | -1 | -1 | 0 | 0 | 122.1 | 114.7 | 106.4 |
| **6** | -1 | 0 | +1 | 0 | 103.4 | 102.7 | 100.6 |
| **7** | +1 | 0 | +1 | 0 | 142.7 | 142.7 | 99.9 |
| **8** | -1 | +1 | 0 | 0 | 135.5 | 136.3 | 99.4 |
| **9** | +1 | +1 | 0 | 0 | 174 | 174.2 | 99.8 |
| **10** | 0 | 0 | 0 | 0 | 152.1 | 152.1 | 100 |
| **11** | 0 | 0 | 0 | 0 | 152.1 | 152.1 | 100 |
| **12** | 0 | +1 | 0 | +1 | 223.6 | 211.5 | 105.6 |
| **13** | 0 | -1 | -1 | 0 | 172.3 | 177.2 | 97.1 |
| **14** | +1 | 0 | -1 | 0 | 211.1 | 210.0 | 100.4 |
| **15** | +1 | -1 | 0 | 0 | 168.5 | 160.5 | 104.9 |
| **16** | 0 | 0 | -1 | +1 | 249.9 | 248.8 | 100.4 |
| **17** | 0 | 0 | +1 | +1 | 177.2 | 179.6 | 98.6 |
| **18** | 0 | -1 | 0 | +1 | 199.3 | 198.9 | 100.1 |
| **19** | -1 | 0 | 0 | -1 | 66.7 | 70.1 | 95.0 |
| **20** | -1 | 0 | -1 | 0 | 168.1 | 166.3 | 101.0 |
| **21** | 0 | 0 | -1 | -1 | 121 | 111.4 | 108.6 |
| **22** | +1 | 0 | 0 | -1 | 87.3 | 90.5 | 96.3 |
| **23** | 0 | +1 | 0 | -1 | 84.3 | 82.9 | 101.6 |
| **24** | -1 | 0 | 0 | 1 | 176.8 | 182.3 | 96.9 |
| **25** | 0 | -1 | +1 | 0 | 98 | 98.3 | 99.6 |
| **26** | 0 | -1 | 0 | -1 | 49.9 | 60.2 | 82.8 |
| **27** | 0 | 0 | 0 | 0 | 152.1 | 152.1 | 100 |

**Table S5:** **Values of estimated regression coefficient and corresponding t-and P-value.**

|  | **Coefficients** | **Standard Error** | **t Stat** | **P-value** |
| --- | --- | --- | --- | --- |
| Intercept | 152.1379 | 4.437377 | 34.28556 | 2.41E-13 |
| **X1** | 20.94488 | 2.218688 | 9.440206 | 6.65E-07 |
| **X2** | 8.825 | 2.218688 | 3.977575 | 0.001834 |
| **X3** | -32.7167 | 2.218688 | -14.7459 | 4.72E-09 |
| **X4** | 66.83654 | 2.218688 | 30.12435 | 1.12E-12 |
| **X1X2** | -1.975 | 3.842881 | -0.51394 | 0.616628 |
| **X1X3** | -0.925 | 3.842881 | -0.2407 | 0.813847 |
| **X1X4** | 10.73463 | 3.842881 | 2.79338 | 0.016242 |
| **X2X3** | 6.725 | 3.842881 | 1.749989 | 0.105621 |
| **X2X4** | -2.525 | 3.842881 | -0.65706 | 0.523545 |
| **X3X4** | -1.9 | 3.842881 | -0.49442 | 0.629936 |
| **X1X1** | 1.559243 | 3.328033 | 0.468518 | 0.64781 |
| **X2X2** | -7.24557 | 3.328033 | -2.17713 | 0.050151 |
| **X3X3** | 1.76693 | 3.328033 | 0.530923 | 0.605159 |
| **X4X4** | -6.47826 | 3.328033 | -1.94657 | 0.075377 |

**Table S6**: Data of Intra-particle diffusion kinetic model for the MB dye adsorption onto SGO@CA composite beads.

| **C_0_(mg/l)** | **Intra-particle diffusion kinetic model** | | | | | |
| --- | --- | --- | --- | --- | --- | --- |
|  | **First step**  **C K R^2^** | | | **Second step**  **C K R^2^** | | |
| 50 | 40.828 | 14.341 | 0.9201 | 71.088 | 3.4821 | 0.8184 |
| 100 | 96.078 | 19.193 | 0.8753 | 107.57 | 14.699 | 0.8756 |
| 200 | 56.636 | 48.563 | 0.9894 | 177.6 | 5.952 | 0.9803 |
| 300 | 128.52 | 27.687 | 0.9965 | 168.31 | 16.204 | 0.9957 |
|  |  |  |  |  |  |  |

**Table S7**: Thermodynamic parameters for the adsorption of MB dye onto SGO@CA composite beads.

| **T(K)** | **ΔGº** | **ΔHº** | **ΔSº** |
| --- | --- | --- | --- |
|  | (KJ/mol) | (KJ/mol) | (J/mol.K) |
| 298 | -28.22 | +3.059 | +94.716 |
| 308 | -29.17 |  |  |
| 318 | -30.117 |  |  |
|  |  |  |  |
